# Quantitative tissue perfusion imaging using nonlinear ultrasound localization microscopy

**DOI:** 10.1038/s41598-022-24986-w

**Published:** 2022-12-19

**Authors:** Jennifer N. Harmon, Zin Z. Khaing, Jeffrey E. Hyde, Christoph P. Hofstetter, Charles Tremblay-Darveau, Matthew F. Bruce

**Affiliations:** 1 Department of Neurological Surgery, University of Washington, Seattle, WA 98105 USA; 2 Philips Medical Systems, Bothell, WA 98041 USA; 3 Applied Physics Laboratory, University of Washington, Seattle, WA 98105 USA

**Keywords:** Biomedical engineering, Acoustics, Imaging techniques

## Abstract

Ultrasound localization microscopy (ULM) is a recent advancement in ultrasound imaging that uses microbubble contrast agents to yield vascular images that break the classical diffraction limit on spatial resolution. Current approaches cannot image blood flow at the tissue perfusion level since they rely solely on differences in velocity to separate tissue and microbubble signals; lower velocity microbubble echoes are removed during high pass wall filtering. To visualize blood flow in the entire vascular tree, we have developed nonlinear ULM, which combines nonlinear pulsing sequences with plane-wave imaging to segment microbubble signals independent of their velocity. Bubble localization and inter-frame tracking produces super-resolved images and, with parameters derived from the bubble tracks, a rich quantitative feature set that can describe the relative quality of microcirculatory flow. Using the rat spinal cord as a model system, we showed that nonlinear ULM better resolves some smaller branching vasculature compared to conventional ULM. Following contusion injury, both gold-standard histological techniques and nonlinear ULM depicted reduced in-plane vessel length between the penumbra and contralateral gray matter (−16.7% vs. −20.5%, respectively). Here, we demonstrate that nonlinear ULM uniquely enables investigation and potential quantification of tissue perfusion, arguably the most important component of blood flow.

## Introduction

Ultrasound is a robust, widely accessible imaging modality with a long history in the measurement of blood flow. Ultrasound has several advantages for clinical and pre-clinical imaging; notably, its real-time capabilities provide a non-invasive, economical tool to assess structural tissue and blood flow changes in longitudinal studies. Newer Doppler approaches have enabled the visualization of lower velocity blood flow flow in smaller vasculature^1^. However, as Doppler shifts from lower velocity blood flow overlap with that of tissue motion, the visualization of blood flow in smaller vasculature including perfusion in the microcirculation (< 1 cm/s in most cases) is lost. To overcome this, contrast-enhanced ultrasound imaging (CEUS) leverages intravenously administered micron-scale gaseous contrast agents to isolate and visualize tissue perfusion^2^. Signal from circulating microbubbles can be isolated from surrounding tissue based on their nonlinear response to ultrasound in a velocity-independent manner, enabling access to blood flow through the entire vascular tree without relying on velocity-based wall filtering approaches. However, CEUS is still subject to trade-offs between penetration depth, sensitivity, and spatial resolution due to the decreasing oscillatory response from microbubbles at elevated transmit frequencies^3^.

Recently, techniques borrowed from optical microscopy have been applied to produce super-resolved vascular maps, breaking the classic “diffraction limit” for spatial resolution ($\sim$100 um at 15 MHz)^4,5^. This approach, termed ultrasound localization microscopy (ULM), is in essence comprised of localization and subsequent inter-frame tracking of spatially-discrete circulating microbubble contrast agents^6^. Super-resolved vascular maps are produced by accumulating these tracks over long acquisition durations (tens of seconds to minutes). Data are typically acquired using plane-wave imaging (commonly $\sim$500–1000 Hz frame rate after angle compounding) in order to improve microbubble tracking and to minimize the total required acquisition duration^7,8^.

In contrast to conventional CEUS, current ULM approaches use linear pulsing schemes, which necessitates spatiotemporal filtering to separate flowing microbubbles from tissue background. However, due to overlap in the velocity of the slowest microbubbles and tissue motion, these bubbles are discarded, preventing investigation of blood flow at the level of tissue perfusion^3,4^. In this work, we overcome this limitation by utilizing a plane-wave, nonlinear pulsing scheme to segment microbubble signals from tissue background independent of velocity^3,9^. We are then able to isolate the slowest moving microbubbles using the singular value decomposition (SVD) to produce ULM images of the microcirculation. Here, we use the rat spinal cord as a model system and contusion injury as a realistic example of pathological hemodynamic disruption. In addition to producing super-resolved images of the microcirculation, quantitative microbubble track parameters (e.g., track length, duration, velocity) represent a novel feature set that can describe the relative quality of microcirculatory flow.

## Results

### Nonlinear ULM outperforms CEUS Doppler in large vessel imaging

**Figure 1:**
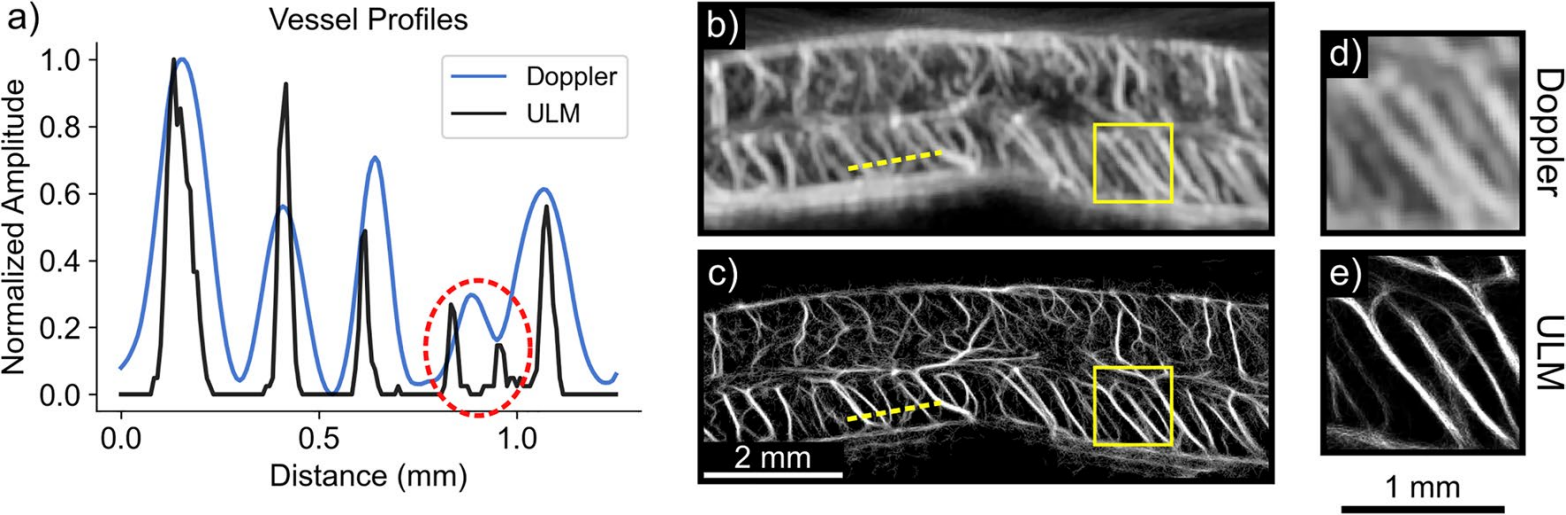
Ultrasound localization microscopy (ULM) improves image resolution compared to contrast-enhanced Doppler imaging. Representative longitudinal images of thoracic levels 8 and 9 of the rat spinal cord are depicted. (**a**) Plots of the normalized amplitude across a subset of vessels (**b**,**c**, dashed yellow line) are shown. (**c**) ULM produces significantly smaller vessel profiles compared to (**b**) contrast-enhanced Doppler imaging (36.1±6.77 um vs. 120±11.2 um, respectively), and distinguishes two separate vessels that overlap in the Doppler image (red ellipse in **a**). Matched zoomed regions illustrate the improved image fidelity in the ULM case (**d**,**e**). Doppler images are displayed with 20 dB dynamic range; ULM density maps are scaled from 0 to 30 detected bubbles.

The spinal cord exhibits both cardiac and respiratory motion even in immobilized and anesthetized rats. To demonstrate the feasibility and utility of ULM in the rodent spinal cord, ULM imaging was compared to nonlinear Doppler imaging^3^. Figure 1 depicts comparative results in the intact thoracic spinal cord (T8–T9). ULM achieved substantially improved separation between adjacent vessels, enabling more accurate quantification of vessel diameter. Vessel diameter was defined as the full width half maximum (FWHM) across the vessel profile; on average, for matched vessels, the diameter obtained from ULM was approximately one third of the diameter obtained from Doppler (36.1±6.77 um vs. 120±11.2 um). Moreover, ULM isolates two vessels which overlapped and were indistinguishable in the Doppler image (Fig. 1a, red ellipse). The full ULM image is shown with a 3-fold increase in spatial resolution compared to the Doppler image (Fig. 1b,c); the zoomed subsection of vessels (Fig. 1d,e) was processed at a 5-fold higher resolution than the Doppler equivalent (5 vs. 25 um per pixel). These results indicate that the accumulated displacements motion correction algorithm applied here is sufficient to enable high quality ULM imaging of the rodent spinal cord.

### Comparison of nonlinear and linear ULM in large vessel imaging

**Figure 2:**
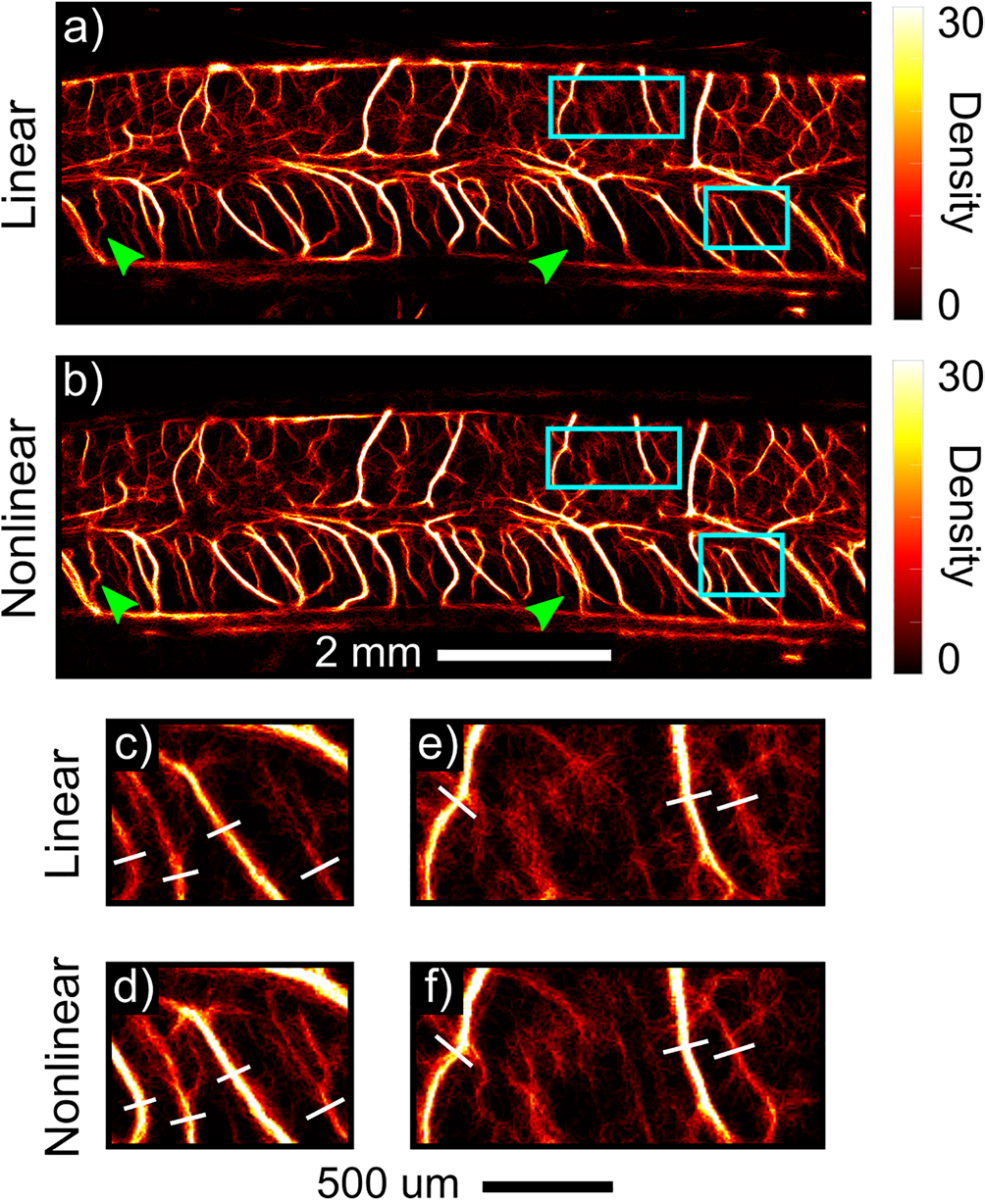
Nonlinear imaging improves sensitivity and resolution compared to conventional ULM. (**a**,**b**) Representative ULM images of thoracic levels 7–8 are displayed. ULM processing was conducted on the same acquisitions, using either the linear (**a**; i.e., all pulses summed) or nonlinear (**b**; i.e., half pulses subtracted for linear signal cancellation) signal components. Green arrows indicate regions with improved sensitivity; this is particularly pronounced in the ventral aspect of the spinal cord. (**c**–**f**) Zoomed images depict representative regions (blue boxes) wherein nonlinear ULM better resolved small vessels (FWHM: 35.0 ± 5.69 um vs. 46.0 ± 8.89 um in **c**–**f**; vessels selected for measurement are marked).

Nonlinear ULM was then compared directly against linear ULM in a large vessel imaging case. Figure 2 depicts a representative example acquired in the intact thoracic spinal cord. Both the linear and nonlinear ULM images are generated from the same acquisitions, either with all pulses summed to preserve the linear signal component (Fig. 2a) or with half-amplitude pulses subtracted for tissue cancellation (nonlinear, Fig. 2b). Nonlinear ULM generally resulted in better resolution, particularly in the smallest vessels. This is most clearly illustrated in the zoomed regions in Fig. 2c–f; nonlinear ULM resolves some vessels which are not resolved with conventional ULM, and those vessels which are resolved in both cases exhibit substantially reduced FWHM in the nonlinear case (35.0 ± 5.69 um vs. 46.0 ± 8.89 um in Fig. 2c–f). This is likely due to reduced localization error attributable to the smaller point spread function of the harmonic signal component as compared to the fundamental. While linear ULM resulted in an excellent depiction of the spinal cord vasculature, nonlinear ULM achieved visually distinct improvements over the conventional method in some regions. These results primarily indicate that conventional and nonlinear ULM are equally capable for large vessel imaging, with a modest advantage in the nonlinear case.

### Eigen-based flow segmentation enables access to tissue perfusion

**Figure 3:**
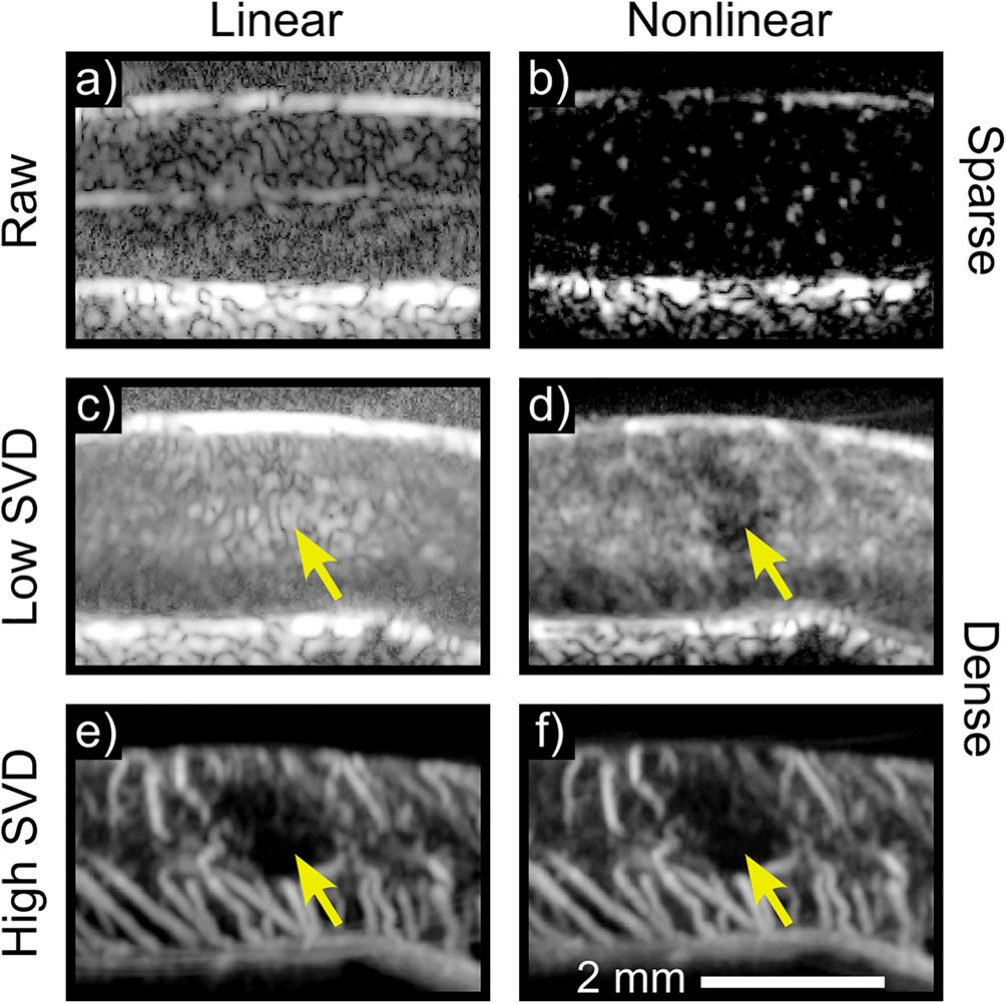
Nonlinear imaging is necessary to describe tissue perfusion. Single frames reconstructed with the linear (**a**) or nonlinear (**b**) signal component are displayed for a representative sparse acquisition (i.e., low microbubble concentration) in the thoracic spinal cord. The linear case solely shows tissue clutter, whereas individual circulating microbubbles are visible in the nonlinear case without any additional filtering or processing. To illustrate the unique utility of nonlinear imaging in describing tissue perfusion, SVD segmented contrast-enhanced Doppler images of a representative dense acquisition (i.e., high microbubble concentration) are displayed. These were acquired following contusion injury. The low projections in the linear case (**c**) are corrupted generally by tissue clutter and more prominently by hematoma in the injury center (yellow arrow). With nonlinear imaging (**d**), tissue clutter and hematoma are cancelled, revealing a perfusion deficit at the contusion center. Both linear and nonlinear imaging (**e**,**f**) can image larger vasculature and associated hypoperfusion following contusion SCI.

**Figure 4:**
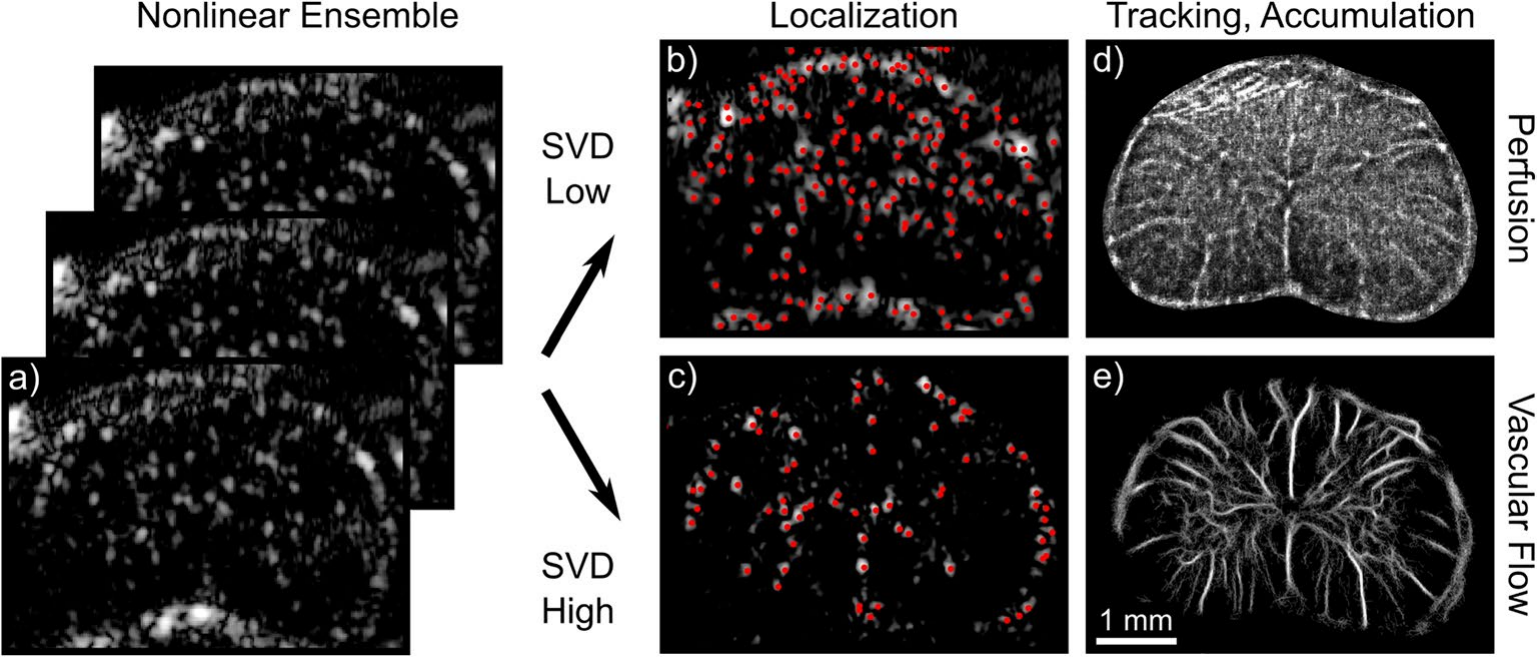
Plane-wave nonlinear imaging enables ULM processing of microcirculatory flow. Amplitude modulated plane-wave ensembles (**a**) are subjected to the singular value decomposition (SVD) to segment and independently analyze flow components based on their relative velocity. By cancelling linear signal components characteristic of tissue clutter, spectral overlap between tissue motion and the slowest moving microbubbles is negated. The lowest projections from the SVD therefore contain signal representing microcirculatory flow, which can be subjected to microbubble localization (**b**) in the same manner as signal from larger vessels (**c**). Following subsequent inter-frame tracking, tracks are accumulated to generate super-resolved images of either tissue perfusion (**d**) or spatially resolved vascular flow (**e**) from the same initial acquisitions. Images were acquired axially at cervical level 5 in the rat spinal cord.

Figure 3 depicts Power Doppler images which illustrate overlap between tissue clutter and microcirculatory flow with linear imaging (Fig. 3a,c); tissue perfusion signal may be recovered by using nonlinear imaging (Fig. 3b,d). By this same principle, the combination of nonlinear imaging and ULM processing provides a novel method for tissue perfusion imaging. The plane-wave amplitude-modulated pulsing scheme utilized for nonlinear ULM resulted in cancellation of the majority of tissue background, which exhibits a primarily linear acoustic response at low MI ($\sim$32-34 dB tissue cancellation)^2,3,9^. The acquired nonlinear ensembles were comprised of three components: non-cancelled tissue (e.g., hematoma due to its high echogenicity at elevated transmit frequencies), circulating microbubbles, and noise. These components were segmented using the SVD. Due to relative differences in decorrelation rates (e.g., velocity), the aforementioned signal components will map to different projection subspaces. To account for the highly echogenic hematoma present following contusion SCI, the first 3 projections were excluded to remove uncancelled tissue and retain only signals from circulating microbubbles. Though this is likely only necessary in the post-injury case, both baseline and post-SCI data were processed in the same manner for consistency. Low velocity sub-spatially resolved projections, 4-35, were attributed to tissue perfusion, whereas projections 125–500 were attributed to spatially resolved vasculature with higher velocity flow^3^. Figure 4 illustrates flow segmentation in the context of the broader ULM processing pipeline and depicts representative examples of ULM on tissue perfusion (Fig. 4d) and vascular flow signals (Fig. 4e). From an application standpoint, Fig. 4 illustrates the utility of ULM in characterizing distinct regions of the spinal cord; images depict cervical level 5 as opposed to thoracic levels 7/8 depicted in Figs. 1, 2, 3. Data were processed in the same manner for both cervical and thoracic acquisitions.

### Tissue perfusion saturates with shorter acquisition times

**Figure 5:**
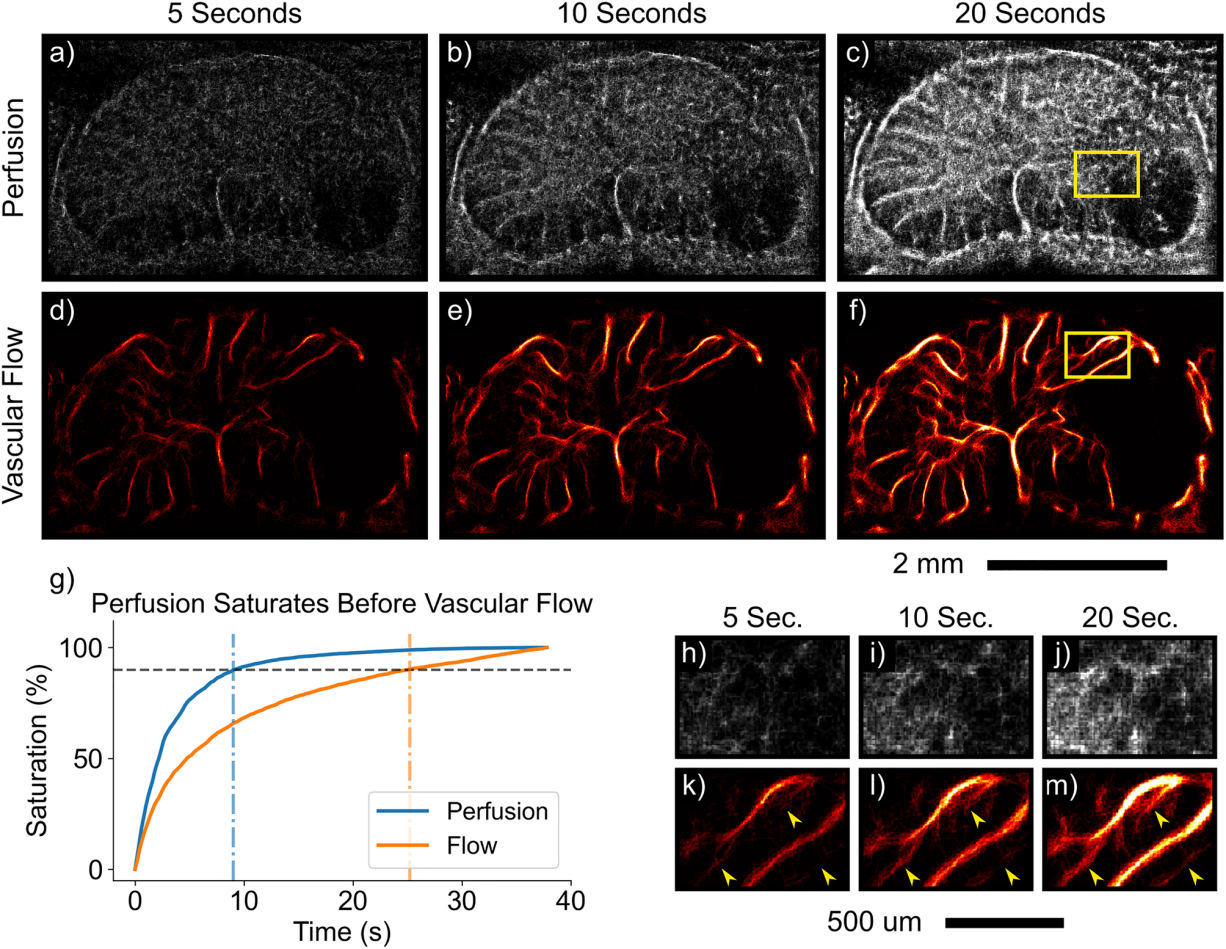
Perfusion ULM imaging is more robust to shorter acquisition times than vascular flow. ULM images depicting increased image fidelity with increasing amounts of input data (5–20 s) are displayed for both tissue perfusion (**a**–**c**) and vascular flow components (**d**–**f**). Representative axial images of the cervical spinal cord, with a hypoperfused region due to a moderate unilateral contusion injury, are depicted. Images are scaled from 0 to 30 detected bubbles. The saturation rate is plotted for a representative example in (**g**). Saturation percent is defined as the number of pixels within the spinal cord filled with a given amount of input data versus the total number of perfused pixels in the spinal cord (e.g., only those pixels which are nonzero after $\sim$40 total seconds total acquisition time). The perfusion image reaches 90% saturation 16.2 s faster than the flow image (9.0 vs. 25.2 s). (**h**-**j**) Zoomed examples of progressive filling of the microcirculation in penumbral tissue adjacent to the contusion and recovery of smaller branching vessels (**k**–**m**) with additional input data are shown.

ULM typically requires long acquisition times, limiting widespread clinical utility. This is particularly true in handheld intraoperative applications with a freely mobile target such as the spinal cord, which exhibits some mobility within the dural sac and is subject to respiratory motion. Images shown in Fig. 5 were acquired at cervical level 5 following a moderate unilateral contusion injury; both perfusion and vascular flow images depict an ischemic deficit on the right side of the spinal cord. To assess the amount of data required to image the microcirculation, the total acquisition time at which output images were 90% saturated was quantified (i.e., signal was observed at 90% of perfused pixels within the spinal cord (Fig. 5g). In terms of data requirements, tissue perfusion saturated before vascular flow (9.0 vs 25.2 s). We hypothesize that this is likely due to the higher relative spatial vessel density in the low rank projections. Given that ultrasound imaging with a linear array transducer produces a 2D projection of a 3D vascular network across a fixed elevational width, a large number of the smallest vessels in the microvasculature overlap spatially when projecting onto the imaging plane. The larger vessels are far more spatially discrete. To support this, the total number of tracks detected in the perfusion projections was 5.6 fold higher than in the flow projections for the case shown in Fig. 5.

### Quantification and display of ULM track data

**Figure 6:**
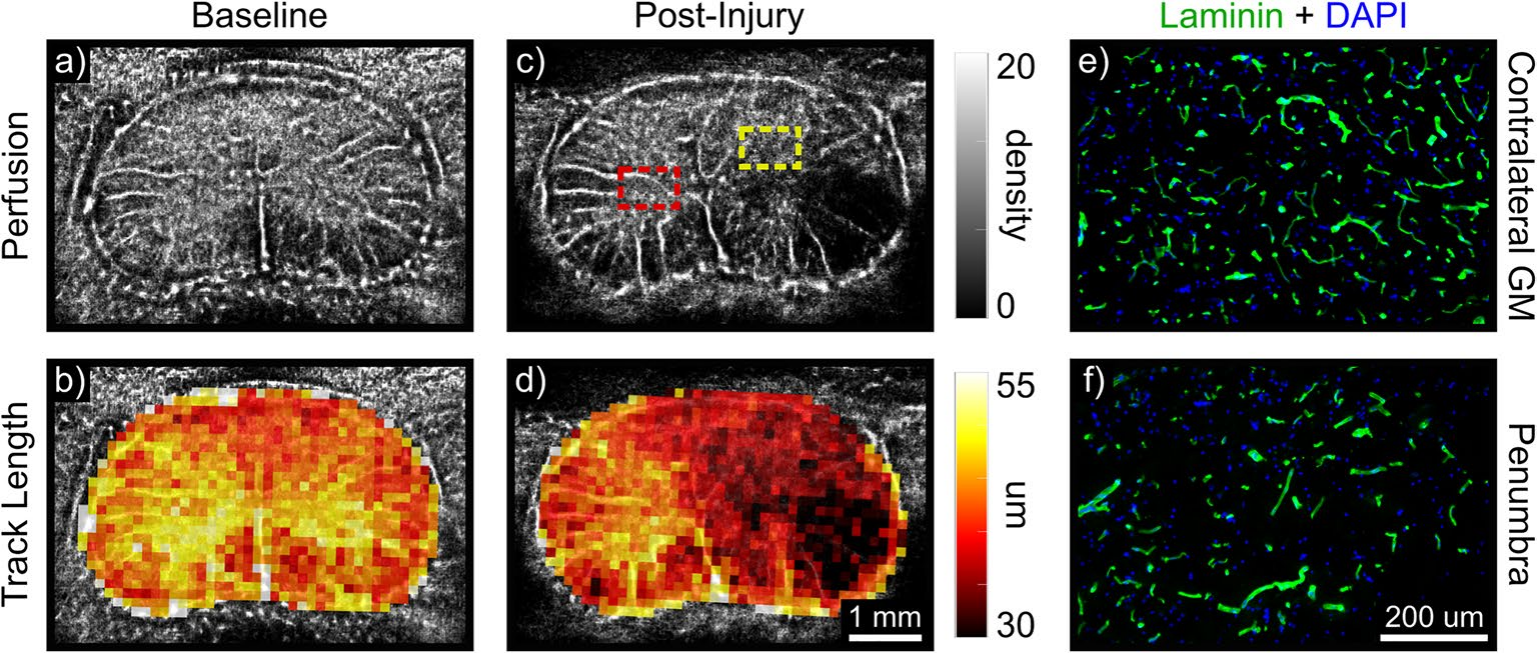
Nonlinear ULM enables novel quantitative analysis and display methods. Perfusion ULM images are depicted at baseline (**a**,**b**) and post unilateral contusion injury (**c**,**d**). Density maps (top) are displayed above quantitative parametric maps (bottom). These parametric maps show the average in-plane travel distance within each bin (in um). These maps recapitulate inherent biological contrast between the gray and white matter at baseline (**b**) and describe pathological disruption of flow in the penumbra following injury (**d**). This agreed with histological analysis (**e**,**f**), which similarly indicated a reduced microvessel density (anti-laminin in green; 504 vs. 283 vessels/mm$^2$) and mean vessel length between the contralateral gray matter and injury penumbra (19.8 ± 1.12 vs 16.5 ± 1.51 um). The corresponding penumbra (dashed yellow) and contralateral gray matter (dashed red) regions are displayed on the ULM image in panel (**c**).

In addition to generating super-resolved density maps and substantially reducing data requirements for image saturation, perfusion ULM enables novel quantitative analysis. Parameters describing the passage of individual microbubbles through a target region can be extracted from track data—for example, the total residence time of a given bubble within the imaging plane, or the track length. Density maps, which inherently provide an absolute estimate of microbubble concentration flowing through the scan plane, are shown alongside parametric maps in Fig. 6(a–d). For these parametric maps, the mean in-plane travel distance (i.e., track length) is shown in each bin. At baseline (Fig. 6a,b), the parametric map recapitulated meaningful biological contrast between the gray matter (center “butterfly”) and white matter (periphery). Following contusion injury, the bubble density image clearly depicted a hypoperfused region on the ipsilateral side (1.44 mm^2^). The corresponding parametric map revealed a gradation in track length between the contralateral gray matter and penumbral tissue adjacent to the hypoperfused region (41.5 ± 3.01 vs. 33.0 ± 2.31 $\upmu$m, respectively; mean ± SD). A gold-standard histological method using anti-Laminin staining Fig. 6(e,f) depicted differences in microvessel density (504 vs. 283 vessels/mm^2^) and mean vessel length (19.8 ± 1.12 vs 16.5 ± 1.51 $\upmu$m) between the contralateral gray matter and penumbra, thereby supporting differences observed in the parametric maps.

## Discussion

Before CEUS was developed, diagnostic ultrasound lacked a contrast agent suitable for microvascular flow measurement present in other diagnostic imaging modalities like contrast CT, contrast MRI, and PET. The development of microbubble contrast agents, along with dedicated imaging techniques, has overcome this barrier and provided ultrasound with a means to image blood flow at the level of tissue perfusion, arguably the component of blood flow having the highest clinical utility. As the location of oxygen and nutrient exchange, quantifying tissue perfusion is of significant experimental and clinical importance. Moreover, it reflects the adaptive response of organs to disease, trauma and malignancy^2^. However, underdevelopment of clinically usable tissue perfusion quantification has resulted in limited use in broader applications.

As illustrated in Fig. 1, ULM represents an extraordinary advancement in spatial resolution over existing Doppler methods, even in comparison to more recent innovations in contrast-enhanced Doppler modalities^3^. However, existing ULM approaches are limited by their use of linear pulsing schemes to image microbubbles, which precludes investigation of the slowest flowing microbubbles at the level of tissue perfusion due to their overlap in velocity with tissue background (Fig. 3), just as in conventional Doppler flow imaging. This necessarily results in an incomplete description of regional microcirculatory hemodynamics. In particular, the inability to investigate and quantify flow at the capillary level is a significant limitation that largely relegates quantification of conventional ULM data to larger vessel anatomy (e.g., tortuosity, diameter) and blood flow velocity. These are not materially different from parameters which may be more rapidly obtained with existing Doppler methods in a less user-dependent manner (e.g., no exogenous contrast, less susceptible to motion). Excellent image quality is likely insufficient to offset the relative impracticality of ULM in many clinical settings. While a number of promising options to reduce acquisition and processing time have been explored, substantive improvements in diagnostic capability will complement these approaches to provide a stronger incentive for clinical adoption^10–12^.

Here, we have developed a novel application of ULM that has utility beyond improving image resolution. Nonlinear plane-wave imaging in combination with eigen-based flow segmentation enables the application of ULM processing to the microcirculation for the first time. In addition to providing high resolution density maps of tissue perfusion, localizing and tracking individual circulating microbubble contrast agents introduces a rich quantitative feature set. In the context of spinal cord injury, the use case explored in the current study, the in-plane travel distance exhibited substantial contrast between penumbral and contralateral tissue. In brief, SCI is comprised of a primary mechanical insult resulting in an ischemic deficit, followed by secondary progressive injury expansion driven by edema, swelling, and inflammation. In this context, identification and quantification of the relative health of penumbral tissue at the acute timepoint is valuable for demarking tissue which may be at risk of further damage during the secondary injury phase. The nonlinear ULM results highlight quantitative microvascular changes that describe hypoperfused penumbral tissue which may be salvageable with acute surgical (e.g., decompression, lumbar drain) or pharmacological (e.g., methylprednisolone) intervention^13–16^. Histological analysis corroborated ULM data, indicating that observed changes in track parameters are grounded in microvascular changes observable with gold standard methods. In particular, a reduction of in-plane travel distance may be descriptive of capillary fragmentation, a well-documented pathophysiological phenomenon following contusion SCI^17–19^.

Nonlinear ULM therefore fills a unique diagnostic niche. Conventional Doppler and ULM imaging are both incapable of quantitatively describing microcirculatory flow. While CEUS bolus kinetics are sensitive to changes in tissue perfusion, they require lengthy acquisition times (minutes) on a single plane which can be difficult and cumbersome to realize clinically, limiting overall clinical use and acceptance. By contrast, nonlinear ULM is robust to variable contrast inflow given that one does not need to capture a dynamic enhancement curve; rather, data can be acquired whenever bubbles are sufficiently sparse to localize and track. While an increased number of tracks within each bin will produce an estimate closer to the true mean, these measures are not directly dependent on either the acquisition time or bubble concentration and may be valuable even with a small number of tracks. Given reduced data requirements in comparison to conventional ULM even without more advanced localization techniques (e.g., $\sim$10 s to achieve 90% saturation), data acquisition for nonlinear ULM is potentially more feasible for clinical use. Moreover, for larger vessels, nonlinear ULM results in modest improvements over conventional ULM in vessel visualization (Fig. 2), likely due to the smaller point spread function characteristic of the harmonic signal as compared to the fundamental. One potential limitation of nonlinear ULM from an acquisition standpoint is the need for additional transmit pulses for the nonlinear pulse sequence; however, this does not materially impact the effective imaging frame rate when using plane wave transmits.

The current method is still limited by lengthy processing times. Motion correction is the rate limiting step (multiple minutes of processing time per second of acquired data); this may be mitigated by migrating to GPU-based processing. The duration of both data acquisition and ULM post-processing may be further shortened by implementing a more specialized microbubble localization algorithm. While the weighted average method applied here achieves fairly rapid subpixel localization prior to upscaling ($\sim$5 s of processing time per second of acquired data), neural network-based approaches have been shown to achieve significantly faster speeds and are more robust to densely populated acquisitions^10,20^.

Nonlinear ULM may suffer from signal to noise ratio (SNR) limitations in high-attenuation situations (e.g., deep abdominal or transcranial imaging). In these cases, conventional ULM may be more sensitive to vessels in deeper regions of the target tissue, or to smaller vessel branches that diverge out of the imaging plane. However, in the context of epidural spinal cord imaging, a realistic intraoperative scenario given routine spinal decompression and stabilization following traumatic injury, the nonlinear component provides more than sufficient SNR for ULM processing.

In order to image the spinal cord post-SCI in this work, the first 3 SVD projections were excluded to remove uncancelled tissue from the highly echogenic hematoma in the vicinity of the hypoperfused region. It is important to note that this limitation is likely specific to acute traumatic applications due to the increased backscatter from the hematoma, and generally will depend strongly on the efficacy of linear signal cancellation^2^. Future work will explore optimal rank threshold selection alongside more complex signal decomposition methods for flow segmentation to retain the maximal amount of low velocity flow and to ensure nonlinear ULM is robust, especially under variable motion conditions.

## Methods

### Ultrasound acquisition sequences for microbubble imaging

Imaging was conducted using a programmable research ultrasound system (Vantage, Verasonics, Kirkland, WA, USA) in conjunction with a 15 MHz linear array transducer (Vermon, Tours, France). A custom amplitude-modulated 5-angle plane wave imaging scheme was used; the implementation and theory supporting this approach have previously been described in detail^3,21^. Details are reproduced here for clarity. Acquisitions consisted of five angle plane wave Doppler imaging (−10 to 10 deg) wherein an amplitude modulated sequence was interleaved on a per angle basis to generate a nonlinear Doppler acquisition (i.e., cancellation of tissue background). After bandpass filtering on receive (centered on 15 MHz), scaling and summation of the amplitude-modulated pulses, and delay-and-sum beamforming, IQ data from each angled transmit were coherently compounded to generate a nonlinear image frame. This was repeated at an effective pulse repetition frequency of 400 Hz to generate a final nonlinear ensemble of 720 frames (1.8 s acquisition duration). For nonlinear ULM, up to 22 total ensembles were acquired for processing (i.e., up to 40 s total acquisition time). Nonlinear Doppler imaging as depicted in Figs. 1 and 3 is described in detail in Bruce et al.^3^. A mechanical index of 0.04 was used to avoid disruption of circulating contrast agents.

### Motion correction

All processing was conducted using Matlab (R2019b, Mathworks, Natick, MA, USA). Tissue-specific motion was isolated by applying the SVD to the linear component of the signal (i.e., half-amplitude pulses were added, not subtracted)^22^. The first 15 lowest velocity projections were retained; these are associated with the highest spatiotemporal coherence, thereby excluding any motion due to circulating contrast agents that may generate spurious displacement estimates. The center of the first ensemble was selected as the global reference frame. Within each ensemble acquisition, the 2D correlation coefficient between each frame and the center frame was calculated. To avoid excessively large displacements between a given frame and the global reference (e.g., peak inhalation vs stationary), displacement estimates were accumulated. Relative reference frames were selected each time the correlation coefficient changed by a set threshold value (0.02). Displacements were estimated between each relative reference frame and were accumulated upstream to the global reference. For any given frame, displacements were then estimated between the frame of interest and the nearest relative reference frame, accumulated, and applied to register the frame of interest to the global reference. For every subsequent ensemble, the center frame was selected as another relative reference frame and registered to the global reference, such that motion correction was done both within and between each ensemble acquisition. This is similar to techniques used in cardiac strain estimation^23^. Displacement fields were estimated and applied to correct the original, nonlinear image ensemble in a non-rigid manner^24^.

### Flow segmentation and pre-processing

Following motion correction, nonlinear ensemble acquisitions were segmented into tissue perfusion and vascular flow signal components using the SVD. Briefly, data were reshaped into a 2D casorati matrix (i.e., one linearized image frame per column), and processed using an implementation of the SVD described in Demené et al.^22^. A hard threshold for delineating which SVD projections comprised perfusion (first singular vectors, high spatiotemporal coherence) and flow signals (last singular vectors, low spatiotemporal coherence) was empirically selected as described in Bruce et al.^3^. Briefly, the rank thresholds for the perfusion and flow subspaces were selected based on Power Doppler images and subsequently applied for all ULM processing. The rank of the perfusion subspace was gradually increased to reduce speckle variance while minimizing any bleed-through from larger, spatially resolved vascular components. The low end cutoff for the flow projections was selected such that larger vessels were clearly visible and spatially incoherent signal from low velocity microcirculatory flow was excluded. Perfusion and flow ensembles were processed separately in subsequent steps to generate independent ULM images. Following flow segmentation, the last 5 projections from the SVD were selected to describe the noise profile. This was smoothed with a Gaussian filter ($\sigma = 20$) and applied to SVD-filtered ensembles to equalize electronic noise across the image depth^25^. A nonlocal means denoising filter was applied along slow-time to reduce spurious bubble localization^26^.

### Microbubble localization and tracking

Different microbubble localization methods were utilized for perfusion and flow ensembles. For perfusion, a weighted average subpixel localization method was applied to the data without any upscaling or additional filtering^20^. This method does not assume the shape of the point spread function. For flow, a cross-correlation based localization method was utilized^26^. Each image was upscaled by a factor of 3 (8x8 um final pixel dimensions) prior to generating a normalized 2D correlation map using an estimated Gaussian point spread function. Images were thresholded (> 0.6 correlation coefficient), and local maxima were identified and recorded as microbubble centers. Following localization, microbubbles were tracked between subsequent frames using a partial assignment algorithm^26–28^. Bubbles were paired using a nearest neighbor approach, wherein a bubble in frame n was paired with the nearest bubble in frame n+1. This is based on the Hungarian assignment algorithm, wherein the total pairing distance is minimized, but is more robust to noise and spurious localizations. In the flow case, microbubble tracks shorter than 7 subsequent frames were excluded to reduce noise in the final image^26^. All tracks were retained in the perfusion case. Tracks were smoothed using a Savitsky–Golay filter (first order, 3 frame window), linearly interpolated by a factor of 10, and resulting positional information was accumulated across all ensemble acquisitions to form microbubble density maps.

### Quantification and parametric maps

Quantitative parameters were extracted from each track to elucidate the dynamics of microbubble passage through the imaging sample volume. Specifically, the total length, duration (e.g., residence time of each microbubble within frame), and average velocity across each track were quantified. Parametric maps were generated to visualize the spatial distributions of these parameters. The spinal cord parenchyma was manually masked based on the microbubble density map. Subsequently, evenly spaced 25 um square bins were defined. The center point of each track was calculated and used to assign tracks to specific bins. For each parameter, the mean value of the distribution was displayed for each bin, overlaid on the microbubble density map. Bins containing fewer than 10 tracks were excluded.

### Rodent spinal cord injury model

All animal procedures were conducted with the approval of the Institutional Animal Care and Use Committee (IACUC) at the University of Washington (Protocols #4362-01 and #4512-01), and in accordance with the ARRIVE guidelines and guidelines from the NIH Office of Laboratory Animal Welfare (OLAW). Female Long Evans rats (12–15 weeks, 200–250 g, Harlan Labs, Indianapolis, IN, USA) were anesthetized using isoflurane (5% induction, 2−3% maintenance, in 1 L/min O_2_). A 24-G tail vein catheter was placed and attached to a three-way stopcock to administer microbubble contrast agents (0.1 mL Definity, Lantheus, Billerica, MA, USA) and subsequent saline flushes (0.2 mL, 0.9% NaCl). The area overlying cervical segment 2 to thoracic segment 2 or thoracic segments 5–11 was shaved, cleaned, and sterilized. Topical analgesic (Lidocaine, 1.5 mg/kg, and Bupivacaine, 1 mg/kg) was administered subcutaneously prior to making a longitudinal incision using a #10 scalpel blade. After subperiosteal dissection of paraspinal muscles, a three-level laminectomy was performed to expose either the cervical (C4–C6) or thoracic (T7–T9) spinal cord for epidural imaging. Animals were stabilized at either the C4 and C6 or T7 and T9 spinous processes during contusion injury, and at either C2 and T2 or T5 and T11 during ultrasound imaging. A 150 kDyn contusion injury was produced at C5 or T8 using an Infinite Horizon impactor (Precision Systems and Instrumentation, LLC, VA, USA). Axial imaging centered over C5, or longitudinal imaging centered at midline in the thoracic case, was conducted at baseline and acutely post-injury (< 1 h). At each timepoint, 15-22 nonlinear ensembles (27–40 s total duration) were acquired starting 4 min after a bolus injection of contrast agent.

### Histology

Following acute image acquisition and euthanasia, animals were perfused with ice-cold PBS (pH 7.4, 200 mL) prior to trans-cardiac perfusion with cold 4% paraformaldehyde (300 mL). A 13 mm segment of the spinal cord corresponding to the window exposed during laminectomy was extracted and post-fixed in 4% paraformaldehyde overnight at 4 °C. Tissue was cryoprotected in a 30% sucrose solution with 0.01% sodium azide prior to freezing for cryosectioning. A cryostat (CM1850, Leica, Wetzlar, Germany) was used to acquire 20 $\upmu$m thick tissue sections which were subsequently thaw-mounted onto gelatin-coated glass slides and stored at −80 °CC. Tissue sections were stained for laminin (L9393, 1:500, Sigma-Aldrich, St. Louis, MO, USA) using established immunohistochemistry protocols to visualize vasculature within the spinal cord tissue. Fluorescent images were acquired using an Axio Zoom V16 fluorescent microscope (Zeiss, Oberkochen, Germany) prior to processing with ImageJ. Quantification of histological images was conducted with a custom Matlab script. Images were thresholded using a Matlab built-in function (imbinarize) prior to skeletonization using an established algorithm.^29^ Vessel counts and anatomical parameters (diameter, length, tortuosity) were derived from the skeletonized data.

## Data Availability

The datasets generated and analyzed during the current study are available from the corresponding author on reasonable request.
